# Two Cases of Lassa Fever Successfully Treated with Ribavirin and Adjunct Dexamethasone for Concomitant Infections

**DOI:** 10.3201/eid2810.220625

**Published:** 2022-10

**Authors:** Sylvanus Okogbenin, Cyril Erameh, Joseph Okoeguale, Osahogie Edeawe, Esele Ekuaze, Kelly Iraoyah, John Agho, Mirjam Groger, Benno Kreuels, Lisa Oestereich, Femi O. Babatunde, Peter Akhideno, Stephan Günther, Michael Ramharter, Till Omansen

**Affiliations:** Irrua Specialist Teaching Hospital, Irrua, Nigeria (S. Okogbenin, C. Erameh, J. Okoeguale, O. Edeawe, E. Ekuaze, K. Iraoyah, J. Agho, F.O. Babatunde, P. Akhideno);; Bernhard Nocht Institute for Tropical Medicine, Hamburg, Germany (M. Groger, B. Kreuels, M. Ramharter, L. Oestereich, S. Günther, T. Omansen);; University Medical Center, Hamburg-Eppenforf, Germany (M. Groger, B. Kreuels, M. Ramharter, T. Omansen);; German Center for Infection Research, Partner Sites Hamburg-Lübeck-Borstel-Riems, Germany (M. Groger, B. Kreuels, L. Oestereich, S. Günther, M. Ramharter, T. Omansen)

**Keywords:** Lassa fever, Lassa virus, viruses, ribavirin, dexamethasone, viral hemorrhagic fever, Lassa mammarenavirus, concomitant infections, Nigeria

## Abstract

Lassa fever is a viral hemorrhagic fever treated with supportive care and the broad-spectrum antiviral drug ribavirin. The pathophysiology, especially the role of hyperinflammation, of this disease is unknown. We report successful remission of complicated Lassa fever in 2 patients in Nigeria who received the antiinflammatory agent dexamethasone and standard ribavirin.

Lassa fever is a viral hemorrhagic fever endemic to West Africa; an estimated 300,000 cases of Lassa fever and 5,000 deaths occur annually (*1*). Lassa fever is currently treated with ribavirin, a nucleoside analog that has broad-spectrum antiviral properties (*2*). Despite being recommended in most national treatment guidelines (*3*,*4*), ribavirin is not formally approved for treatment of Lassa fever. Evidence supporting a beneficial effect of ribavirin for treatment of Lassa fever is scarce, and the pivotal landmark study (*2*) showed a high risk for bias (*5*,*6*). A study focused on evaluation of pharmacokinetics of ribavirin to better characterize its role in treatment for Lassa fever (*7*). Severe Lassa fever with lethal outcome is associated with encephalopathy, acute kidney injury, and respiratory failure (*8*). Lassa virus (LASV) has been observed in the cerebrospinal fluid of patients exhibiting clinical symptoms of encephalitis (*9*,*10*). However, it is insufficiently understood how LASV causes pathology; in severely ill patients, mediators of coagulation, as well as inflammatory markers, were found to be dysregulated (*11*).

Because of the epidemic potential of Lassa fever and its high case-fatality rates in hospitalized patients, Lassa fever was added to the World Health Organization blueprint priority list of diseases for research and development, urging intensified research, including improved treatments. Based on the excess in inflammatory response observed in severe Lassa fever patients (*12*), host directed antiinflammatory treatment has been discussed as adjunct therapy. This therapy has been found to be beneficial, as indicated by expert’s opinion in LASV-infected adults and pregnant women.

We report 2 patients, 1 who had severe Lassa fever complicated by COVID-19 and acute kidney injury (patient A), and 1 who had acute kidney injury and neurologic complications (patient B) who, in addition to intravenous ribavirin and supportive care, received dexamethasone. Dexamethasone is a glucocorticoid agent also successfully used for other severe viral infections, such as COVID-19, to reduce damage conferred by systemic hyperinflammation (*13*).

## The Study

Patient A was a 72-year-old man who came to Irrua Specialist Teaching Hospital (ISTH) in Irrua, Nigeria, on January 15, 2021, because of reported weakness and poor appetite. He had previously been given artemether/lumefantrine for suspected malaria for 4 consecutive days. However, his symptoms persisted. His medical history included diabetes mellitus type 2 and arterial hypertension. At examination, the patient was in apparent good clinical condition, afebrile, and had normal vital signs and adequate peripheral oxygenation at ambient air.

A molecular test of an oropharyngeal swab specimen for SARS-CoV-2 showed a positive result (reverse transcription PCR [RT-PCR] cycle threshold [Ct] 14.97 for betacoronavirus] and Ct 12.3 for SARS-CoV-2). Oral dexamethasone therapy was initiated, and the patient was referred to domestic quarantine, according to standard local practice. Two days later, the patient came again to the hospital and reported progressive worsening of body weakness; he appeared pale and in a markedly reduced clinical condition.

 He had a body temperature of 38.0°C, heart rate 105 bpm, blood pressure 115/75 mm Hg, respiratory rate 24/min, and 95% oxygen saturation in ambient air. Auscultation of the chest and further physical examination showed no abnormalities.

The patient was admitted to the isolation ward and tested for Lassa fever because of persistent fever and worsening condition in a zone to which Lassa fever is endemic. The result was positive (Ct 33.33, by RealStar Lassa Virus RT-PCR Kit; Altona Diagnostics, https://www.altona-diagnostics.com).

During hospitalization, the patient required 2–4 L/min of supplemental oxygen by nasal cannula, received intravenous ribavirin according to the Irrua regimen (*3*) and continued therapy with dexamethasone (8 mg orally every 8 h). Laboratory diagnosis showed anemia and acute renal failure (Table). An initial treatment with prophylactic low molecular weight heparin was discontinued once Lassa fever was diagnosed to avoid exacerbation of risk for bleeding in viral hemorrhagic fever. The patient had progressive normocytic anemia and received 2 blood transfusions. Stool for occult blood was negative, and there were no further clinical signs of external or internal bleeding.

**Table:** Laboratory parameters of 2 patients in Nigeria at admission who had Lassa fever, were given ribavirin and dexamethasone, and showed favorable outcomes*

Parameter	Patient A	Patient B
Hematology		
Hemoglobin, g/dL	9.8	9.2
Packed cell volume, %	26.40	31.40
Erythrocytes, × 10^9^ cells/L	3.04	3.77
Mean corpuscular volume, fL	88.5	83.3
Mean corpuscular hemoglobin, pg	32.2	24.4
Mean corpuscular hemoglobin concentration, g/dL	36.4	29.3
Red cell distribution width, %	29.2	27.4
Platelets/mm^3^	155,000	140,000
Mean platelet volume, fL	12.4	10.3
Platelet distribution width, %	28.1	27.4
Total leukocytes, x 10^9^ cells/L	7,800	14,400
Neutrophils, %	66.40	NA
Lymphocytes, %	18.50	NA
Monocytes, %	12.40	NA
Eosinophils, %	2.40	NA
Basophils, %	0.30	NA
Clinical chemistry		
Creatinine, mg/dL	2.8	1.1
Urea, mg/dL	81	57
Bilirubin, mg/dL	0.8	0.6
Total protein, g/dL	6.8	7.8
Alanine aminotransferase, IU/L	6	11
Aspartate aminotransferase, IU/L	19	14
Electrolytes		
Sodium, mmol/L	136	NA
Potassium, mmol/L	5	NA

*NA, not available.

Over time, the condition of the patient improved, and the creatinine level returned to within the reference range. The patient had sufficient urinary output and did not require dialysis. On day 21 after admission, repeat PCR testing results for SARS-CoV-2 and LASV were negative, and all vital signs were stable. The patient was discharged in good health.

Patient B was a 40-year-old woman who was referred by a peripheral healthcare center to ISTH on March 15, 2021. She came to the hospital because of fever, headache, and loss of appetite. She was empirically given artemether/lumefantrine but symptoms persisted. This finding prompted testing for LASV at ISTH; result was positive (Ct 31.80, by RealStar Lassa Virus RT-PCR Kit).

She had a body temperature of 37.8°C, heart rate (tachycardia) 118 bpm/min, blood pressure 89/69 mm Hg, regular respiration, and 99% oxygen saturation in ambient air. Physical examination showed no additional major pathologic findings. 

Antiviral therapy with intravenous ribavirin according to the Irrua regimen was initiated. On day 3 of admission, the patient reported intense headaches and had persistent fever of 38.3°C. Examination showed pronounced neck stiffness that was interpreted as a sign of meningeal irritation. Lumbar puncture was not performed to minimize risk for bleeding in the context of a viral hemorrhagic fever with unknown mechanisms of bleeding and lacking availability for coagulation testing.

Based on a clinical diagnosis of meningitis, the patient was empirically given intravenous ceftriaxone (2 g every 12 h) and intravenous dexamethasone (4 mg every 12 h). Over the next few days, fever and other complaints subsided gradually and vital signs returned to reference ranges. Ceftriaxone and dexamethasone were discontinued after 7 days of treatment. On day 13 of admission, headache and neck stiffness were markedly reduced, and ribavirin was switched to oral therapy. PCR for LASV on day 14 of admission was negative. Ribavirin was then discontinued, and the patient was discharged.

Both patients were seen for follow-up. They appeared to be in good health and recovered without sequelae.

## Conclusions

Improved treatment options for Lassa fever are urgently needed because convincing evidence for use of ribavirin is lacking (*5*,*6*). Hyperinflammation is a consistent feature in severe Lassa fever (*11*,*12*), as well as in other severe viral infections. Because corticosteroids in general, and dexamethasone as a particularly potent derivative, rapidly reduce hyperinflammation, their use might address this pathophysiologic process. Fear of exacerbation of viral replication and thus worsening the outcome of Lassa fever has so far prevented its routine use for Lassa fever.

In the 2 cases reported, dexamethasone was initiated for treatment of concomitant infections, rather than for indication of Lassa fever. No apparent detrimental effects on the clinical course of disease or virologic or laboratory features were observed. Both patients were severely ill, yet recovered after receiving dexamethasone and ribavirin therapy and showed no sequelae. Patient A had considerable risk factors for poor disease outcome of Lassa fever, such as preexisting chronic concurrent conditions and co-infection with COVID-19. The disease course was complicated by anemia and acute kidney injury. Treatment with dexamethasone might have also contributed to reducing hyperinflammation causing or contributing to these pathologies and similarly to the clinical signs of meningeal inflammation in patient B.

Although these 2 cases of severe Lassa fever cannot be interpreted as proven evidence of the benefit of dexamethasone in Lassa fever therapy, we have not observed any considerable side effects, which provides reassurance for safe assessment in future interventional clinical trials. We suggest that future systematic research into the exact pathophysiology of Lassa fever and a systematic clinical investigation of antiinflammatory and immune-modulatory drugs, such as dexamethasone, for ancillary therapy for Lassa fever are warranted.

## References

[1] Kenmoe S, Tchatchouang S, Ebogo-Belobo JT, Ka’e AC, Mahamat G, Guiamdjo Simo RE, et al. Systematic review and meta-analysis of the epidemiology of Lassa virus in humans, rodents and other mammals in sub-Saharan Africa. PLoS Negl Trop Dis. 2020;14:e0008589. 10.1371/journal.pntd.0008589 32845889 PMC7478710

[2] McCormick JB, King IJ, Webb PA, Scribner CL, Craven RB, Johnson KM, et al. Lassa fever. Effective therapy with ribavirin. N Engl J Med. 1986;314:20–6. 10.1056/NEJM198601023140104 3940312

[3] Nigeria Center for Disease Control. National Guideline for Lassa Fever Case Management. 2018 [cited 2022 Feb 2]. https://ncdc.gov.ng/themes/common/docs/protocols/92_1547068532.pdf

[4] World Health Organization. Clinical management of patients with viral haemorrhagic fever: a pocket guide for front-line health workers: interim emergency guidance for country adaptation. 2016 [cited 2022 Feb 2]. https://apps.who.int/iris/bitstream/handle/10665/205570/9789241549608_eng.pdf

[5] Eberhardt KA, Mischlinger J, Jordan S, Groger M, Günther S, Ramharter M. Ribavirin for the treatment of Lassa fever: A systematic review and meta-analysis. Int J Infect Dis. 2019;87:15–20. 10.1016/j.ijid.2019.07.015 31357056

[6] Salam AP, Cheng V, Edwards T, Olliaro P, Sterne J, Horby P. Time to reconsider the role of ribavirin in Lassa fever. PLoS Negl Trop Dis. 2021;15:e0009522. 10.1371/journal.pntd.0009522 34237063 PMC8266111

[7] Erameh C, Edeawe O, Akhideno P, Eifediyi G, Omansen TF, Wagner C, et al. Prospective observational study on the pharmacokinetic properties of the Irrua ribavirin regimen used in routine clinical practice in patients with Lassa fever in Nigeria. BMJ Open. 2020;10:e036936. 10.1136/bmjopen-2020-036936 32303517 PMC7200043

[8] Duvignaud A, Jaspard M, Etafo IC, Gabillard D, Serra B, Abejegah C, et al.; LASCOPE study group. Lassa fever outcomes and prognostic factors in Nigeria (LASCOPE): a prospective cohort study. Lancet Glob Health. 2021;9:e469–78. 10.1016/S2214-109X(20)30518-0 33740408 PMC7970450

[9] Okokhere PO, Erameh CO, Alikah F, Akhideno PE, Iruolagbe CO, Osazuwa OO, et al. Acute Lassa virus encephalitis with Lassa virus in the cerebrospinal fluid but absent in the blood: a case report with a positive outcome. Case Rep Neurol. 2018;10:150–8. 10.1159/000490374 30057542 PMC6062684

[10] Günther S, Weisner B, Roth A, Grewing T, Asper M, Drosten C, et al. Lassa fever encephalopathy: Lassa virus in cerebrospinal fluid but not in serum. J Infect Dis. 2001;184:345–9. 10.1086/322033 11443561

[11] Strampe J, Asogun DA, Speranza E, Pahlmann M, Soucy A, Bockholt S, et al. Factors associated with progression to death in patients with Lassa fever in Nigeria: an observational study. Lancet Infect Dis. 2021;21:876–86. 10.1016/S1473-3099(20)30737-4 33484646 PMC8212868

[12] Port JR, Wozniak DM, Oestereich L, Pallasch E, Becker-Ziaja B, Müller J, et al. Severe human Lassa fever is characterized by nonspecific T-cell activation and lymphocyte homing to inflamed tissues. J Virol. 2020;94:e01367–20. 10.1128/JVI.01367-20 32817220 PMC7565638

[13] Horby P, Lim WS, Emberson JR, Mafham M, Bell JL, Linsell L, et al.; RECOVERY Collaborative Group. Dexamethasone in hospitalized patients with COVID-19. N Engl J Med. 2021;384:693–704. 10.1056/NEJMoa2021436 32678530 PMC7383595

